# Carbon Nanotube-Supported Dummy Template Molecularly Imprinted Polymers for Selective Adsorption of Amide Herbicides in Aquatic Products

**DOI:** 10.3390/nano13091521

**Published:** 2023-04-29

**Authors:** Sili Zeng, Chenhui Li, Li Huang, Zhongxiang Chen, Peng Wang, Dongli Qin, Lei Gao

**Affiliations:** 1 Heilongjiang River Fisheries Research Institute, Chinese Academy of Fishery Sciences, Harbin 150070, China; 2 College of Fisheries and Life Science, Shanghai Ocean University, Shanghai 201306, China; 3 Supervision, Inspection and Testing Center for Fishery Environment and Aquatic Products (Harbin), Ministry of Agriculture and Rural Affairs, Harbin 150070, China; 4 Key Laboratory of Control of Quality and Safety for Aquatic Products, Ministry of Agriculture and Rural Affairs, Beijing 100141, China; 5 College of Chemistry, Chemical Engineering and Resource Utilization, Key Laboratory of Forest Plant Ecology, Northeast Forestry University, 26 Hexing Road, Harbin 150070, China

**Keywords:** carbon nanotubes, MSPD, aquatic products, amide herbicide

## Abstract

In this study, a carbon nanotube (CNTs)—supported dummy template molecularly imprinted polymer (DMIPs) material was synthesized and utilized for the detection of amide herbicides in aquatic products via matrix solid-phase dispersion technology (MSPD). The DMIPs material was characterized, and its adsorption kinetics and isotherm were determined, the adsorption model was established, and the selective adsorption coefficient was calculated. The extract parameters of the method were optimized and successfully employed for the separation, analysis and detection of real samples, with satisfactory detection limits and linear ranges obtained. By comparing with other methods, the CNTs@DMIPs combined with MSPD technology established in our study can effectively solve false negative problems caused by insufficient destructive force, using dummy template molecules can also address the issue of false positives caused by template molecule leakage in molecular imprinting. Overall, the method is appropriate for the separation and detection of endogenous substances from highly viscous and poorly dispersed samples and is used as a routine detection tool in the aquaculture industry.

## 1. Introduction

The use of herbicides is a widespread agricultural practice to reduce the impact of weeds on crops [1]. Among the many herbicides, amide herbicides are widely used as rice herbicides in China due to their high efficacy and selectivity against a large variety of weeds. With the promotion of the rice-fishing interaction model in my country, herbicides have the risk of entering the fish. However, most of the amide herbicides are harmful for human and fish. Butachlor could cause changes in histopathologically and certain haematological parameters in the kidneys of rainbow trout [2]. At relatively high concentrations, the combination of bensulfuron and acetyl chloride can cause behavioral and morphological effects on juvenile *P. clarkia* [3]. Herbicides can accumulate and pose a health threat to humans through the amplification of the food chain. Therefore, it is very important to determine and monitor these residues in fish.

Based on this, some separation methods such as gas chromatography (GC) and liquid chromatography (LC) were used to separate of amide herbicides. Current separating methods include GC, LC and various sensitive detectors such as electron-capture detection (ECD) [4], mass spectrometry (MS) [5], or tandem MS [6,7,8,9,10]. The detection of trace levels of herbicides in fish species is variable. A number of tests have been reported to detect aquatic products. Generally, the separation time required by gas chromatography is usually longer than that used by liquid chromatography, and the qualitative effect of mass spectrometry is usually due to other detectors. Therefore, we chose liquid chromatography-tandem mass spectrometry (LC-MS/MS) technology as the detector.

Effective pre-treatment often leads to better test results. Frequently used methods include microwave-assisted extraction (MAE) [11], solid-phase extraction (SPE) [12,13,14], solid-phase micro-extraction (SPME) [15,16], quick, easy, cheap, effective, rugged, and safe (QuEchERS) [17] and matrix solid-phase dispersion (MSPD) [18]. Optimization of the traditional sample pre-treatment method has resulted in a significant increase in sensitivity, but its lack of selectivity and specificity has led to strong matrix effects. Prior to LC-MS/MS analysis, it is desirable to develop a technique that allows for highly selective extraction and enrichment of amide herbicides in more complex matrices.

MSPD is a pre-treatment technique, mainly used for solid samples such as animal tissues, that combines sample homogenization, destruction, extraction, and purification with the advantages of practicability, versatility, high-throughput, low cost-effectiveness and speed, and has been used to extract residues of amide herbicides from different complex matrices [19]. The crushing process is designed to separate the sample into the smallest possible pieces so that the surface area is large and can be fully extracted. This extraction method is especially suitable for matrix like fish. The organic matter in the fish sample can be extracted by thorough grinding and complete dispersion. However, there are few reports in the literature on the use of MSPD for the analysis of amide herbicide residues in fish samples and the main disadvantage of conventional SPEs is their low selectivity [20]. Molecularly imprinted polymers(MIPs) are polymeric materials with synthetic binding sites co-polymerized with functional monomers, template molecules and cross-linkers and are widely employed for the pretreatment and selective enrichment of target compounds in complex matrices [21,22]. Based on this, completely non-covalently interacting molecular imprints were introduced into our synthetic polymers. Molecular imprinting technology can design specific adsorption materials for different small molecules to be tested according to experimental requirements [23].

Carbon nanotubes have been applied in different fields because of the numerous unique properties of this kind of material. It was applied in fishery and environmental samples detection [24], biosensing application (for example, enzymes and biological molecules) [25]. Generally, CNTs are divided into single-walled and multi-walled. Multi-walled carbon nanotubes (MW-CNTs) have unique advantages in conductivity, thermal conductivity, and mechanical strength [26]. They have a large specific surface area, low density [27], and can be encapsulated in MIPs, greatly improving their adsorption capacity [28,29]. Despite the high specific surface area of MW-CNTs, their lack of selectivity can become a bottleneck for their application. Therefore, we developed an adsorption and analysis scheme for amide herbicides, performed chemical modification on the surface of MW-CNTs by wrapping them with MIPs layers to address the issue of non-selective adsorption.

Furthermore, one of the primary challenges in applies in MIPs for MSPD is the leakage of residual template molecules. In the previous research process of our research group, it was found that using traditional sample pretreatment techniques can lead to issues such as false negatives and false positives [30]. Even minimal leakage can significantly affect the experimental result when MIPs are used to separate and enrich samples, in particular when analyzing trace samples. To overcome this issue, we employ the use of dummy template molecules that have a chemical structure that is comparable to that of the target substance. So long as two dummy molecules can be isolated, any leakage of the dummy template will have no effect on the analytical results. Deschlorometolachlor was one of the metolachlor metabolites, MSPD with a new adsorbent based on a carbon nanomaterial and MW-CNTs were used to be supporter.

To summarize, the experiment involved combining MIPs with MW-CNTs and employed deschlorometolachloras template molecules to synthesize CNTs@DMIPs as MSPD sorbent. CNTs@DMIPs has high selectivity and adsorption capacity. Contamination by amide herbicides is reduced by using deschlorometolachlor as a template molecule. The focus of this research is to develop and validate an effective, convenient, and time-saving method for the simultaneous analysis of amide herbicides in fish. The method consists of MSPD-cleaning and subsequent LC-coupling with MS-MS. To our knowledge, this work is the first to report the production of MIPs capable of identifying amide herbicides, using deschlorometolachlor as a dummy template. This material was used as an MSPD dispersant to isolate amide herbicides from fish samples. This method combines the selective recognition of MIPs for amide herbicides with their application in aquatic products. In this experiment, a MSPD method was established and satisfactory results were obtained with aquatic products.

## 2. Materials and Methods

### 2.1. Chemicals Reagents and Standard

CNTs were bought from Shenzhen Nanoport (Shenzhen, China) (purity ≥ 95%). Azobisisobutyronitrile, nitric acid (65–68 wt%), methanol, acrylamide and chloroform were obtained from Guangfu (Tianjin, China), acetochlor, alachlor pretilachlor, metolachlor, butachlor, dimethachlor, diethatyl ethyl and deschlorometolachlor were acquried by Dr. Ehrenstorfer (Augsburg, Germany). Ethylene glycol dimethacrylate (EDGMA), Methacrylic acid (MAA), hydrochloric acid, acetic acid, sulfoxide chloride and dimethylformamide were of analytical grade and supplied by Kermel (Tianjin, China).

Liquid chromatography-mass spectrometry (LC-MS) grade NH_4_OAc, acetonitrile and methanol were provided by Fisher (Pittsburgh, PA, USA). A Milli-Q Water System (Millipore, Billerica, MA, USA) has been used to supply high purity water. All other reagents used were of chromatographic grade.

### 2.2. Standard Solution Preparation

The amide herbicide standard stock solutions (metolachlor, acetochlor, alachlor, butachlor, pretilachlor, diethatyl ethyl, and dimethachlor) were produced in methanol at a concentration of 100 μgmL^−1^. All of the stock solutions were stored at 4 °C. Freshly prepared working standards solutions are obtained by diluting appropriate amounts of the above solutions.

### 2.3. Preparation of Fish Samples and Spiked Samples

Fish samples used to develop the method were sourced from the local market, located in Harbin, China. The blank fish sample selected for validation was the one fish sample (*Cyprinus carpio*) that was found to be free of amide herbicides by the national standard method used in China. (No.26, acetochlor; No. 45, pretilachlor; No. 270, alachlor; No. 395, metolachlor. GB/T 20772–2008). Spiked samples were prepared by addition of amide herbicide to the blank fish sample and spiked samples were left at room temperature for 30 min.

### 2.4. Preparation of CNTs@DMIPs

The carbon nanotube molecularly imprinted dummy templates (CNTs@DMIPs) have been prepared following the literature method with some modifications [31,32]. HNO_3_ solution (100 mL) at 80 °C for 6 h was used to remove impurities such as metallic catalysts in the CNTs (0.5 g).CNTs were subsequently washed with ultrapure water. Next, in a round-bottomed flask at 70 °C for 12 h, the activated CNTs were reacted with sulphoxide chloride (50 mL). The mixture was distilled at 90 °C to remove the sulphoxide chloride. The acylating CNTs were then dried under vacuum at 60 °C for an overnight period. Then, 100 mL of dimethylformamide was added to the desiccated acylating CNTs and 6.0 g of acrylic amide. After an ultrasonic treatment for 10 min, the reaction was carried out for 24 h at a temperature 45 °C. Hydrochloric acid (0.12 mol L^−1^) and water were used to wash the vinyl functionalized CNTs. CNTs (0.15 g), deschlorometolachlor (1 mmol), MAA (4 mmol), EDGMA (40 mmol) and initiator azobisisobutyronitrile (0.5 g) were dissolved in 40 mL of chloroform in a beaker. In a 250 mL three-necked bottle, 2 g of polyvinyl alcohol was mixed with 200 mL of water under heating. After allowing the mixture to cool to room temperature, slowly pour the entire mixture in the small flask into the three-necked bottle. The mixture was allowed to react for 6 h in a water bath at 60 °C and then allowed to react for 15 h at room temperature with stirring. Upon completion of the reaction, the polymers were employ to Soxhlet extraction with methanol:acetic acid (9:1, *v*/*v*) to extract the template. The CNTs@DMIPs was dried overnight at 60 °C under vacuum. With the exception of the addition of the template molecule, non-imprinted polymers (CNTs@DNIPs) were synthesized under similar conditions. The prepared CNTs@DMIPs was characterized by means of transmission electron microscopy (TEM) and Fourier Transform Infrared (FTIR).

### 2.5. Sample Extraction and Clean-Up

The edible parts of the fish were removed and ground using a mincer. The fish (0.2 g) was placed in an agate mortar and gently mixed with 0.2 gCNTs@DMIPs for 5 min using a pestle to acquire a homogeneous mixture. The homogenised sample was then transferred to a cassette (5 mL pre-made frames, polypropylene tubes), which was pre-packed at 20 mm porosity. Elute with 5.0 mL acetonitrile-acetic acid (95:5, *v*/*v*) at a flow rate of 1.0 mL min^−1^ after rinsing with 3.0 mL 10 % aqueous methanol. Evaporate to dryness under nitrogen gas at 40°C and redissolve the residue with 0.5 mL of aqueous acetonitrile, and then filtrated by a filter membrane of 0.22 μm for further LC-MS/MS analysis.

### 2.6. Selective Adsorption of AmideH

**Figure 1:**
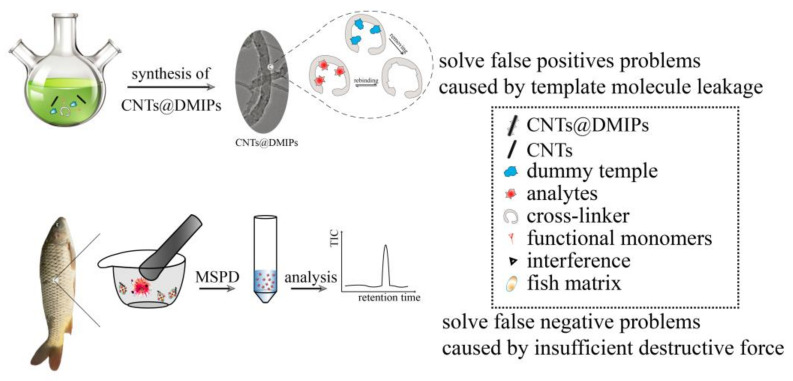
The whole fabrication steps and diagrammatic illustration for the CNTs@DMIPs preparation and application process.

CNTs@DMIPs completes the manufacturing steps and testing process as shown in Figure 1. The recognition characteristics of CNTs@DMIPs for amide herbicide were investigated. The binding experiment was performed by adding 30 mg of CNTs@DMIPs or CNTs@DNIPs to 5 mL working liquid (amide herbicide or atrazine). The centrifuge tube was then stirred for a predetermined time. The concentrations of these standard solutions were all 50 mg L^−1^. After adsorption equilibrium was reached, the supernatant was obtained by centrifugation and the concentrations of amide herbicides or atrazine in the remaining solutions were analyzed by liquid chromatographic-triple quadrupled tandem mass spectrometry (LC-QqQ MS).

### 2.7. LC-QqQMS

Chromatographic separation was carried out on a Waters UPLC H-Class separation unit with autosampler, quaternary solvent delivery pump and Waters Acquity BEH C_18_ 1.7 µm (2.1 × 50 mm) analytical column. Mass spectrometry was performed in a triple quadrupole mass spectrometer (Xevo TQS, Waters) with an electrospray ionisationsource (ESI) interface. Chromatographic was performed using a gradient profile with mobile phase A (5 mmol L^−1^ NH_4_OAc/0.1% formic acid/water) and mobile phase B (acetonitrile). The elution gradient was 30% B, 1 min held, then up to 5% B in 1 min, 1.5 min held, and then back to 10% B in 0.1 min, 1.4 min held to equilibrate the column. The whole process took 5 min. The volume of injection was 10 μL, the temperature of column was controlled at 30 °C and flow rate was set at 0.3 mL min L^−1^, MS conditions were: capillary, 3.0 kV; source offset 30 V; desolvation gas, 800 L hr^−1^; desolvation temperature, 450 °C; cone gas, 250 L hr^−1^; nebulizer, 7.0 Bar; collision gas flow, 0.14 mL min^−1^. source temperature, 150 °C; multiple reaction monitoring (MRM) transitions for target compounds were displayed in the following way, acetochlor(34256-82-1; 270.0 →224.1*,10; 270.0 →148.1,18), pretilachlor(51218-49-6; 312.1 →252.1*,15; 312.1 →176.2,28), alachlor(15972-60-8; 270.0 →238.1*,11; 270.0 →162.1,19), butachlor(23184-66-9; 312.1 →238.1*,11; 312.1 →162.2,23), metolachlor(51218-45-2; 284.1 →252.1*,15; 284.1 →176.2,25), diethatylethyl(38727-55-8; 312.1 →238.2*,26; 312.1 →162.2,16), dimethachlor(50563-36-5; 256.1 →224.1*,14; 256.1 →148.2,24). Besides, atrazine was as the recognition molecule (216.1 →174.1, 17; 216.1 →132.1,22). Deschlorometolachlor was as the dummy template molecules (250.2 →176.3, 22; 250.2 →218.2, 15).

### 2.8. Data Analysis

LC-QqQMS, Data acquisition was done working in MRM mode. Masslynx software (version 4.1) was employed for instrument control, analysis and data collection.

## 3. Results

### 3.1. Characterization Results

**Figure 2:**
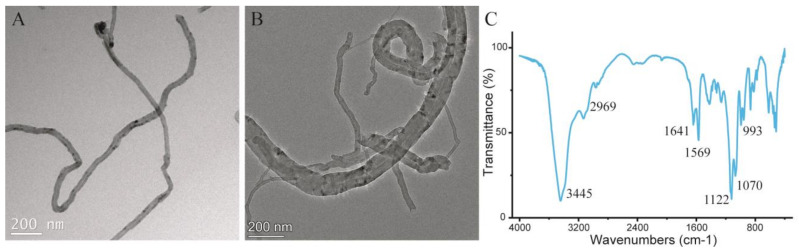
The TEM image of CNTs (**A**) and CNTs@DMIPs (**B**); FT-IR spectra of CNTs@DMIPs (**C**).

Figure 2A,B display TEM images of CNTs and CNTs@DMIPs, respectively. The differences in the morphological features were shown. DMIPs were successfully deposited on CNTs surfaces. The imprinted polymer layer was approximately 28 nm thick. Comparing CNTs and CNTs@DMIPs, it could be concluded that CNTs@DMIPs were successfully synthesized, which increased the surface porosity of DMIPs and enhanced their adsorption capacity. Figure 2C shows FT-IR spectra of CNTs@DMIP, the peak at 3445 cm^−1^ attributed to the stretching vibration of OH groups. The stretching modes of C-H were related to the peak at 2969 cm^−1^. The peak at 1641 cm^−1^ demonstrated that the modified CNTs@DMIPs surface was successful in grafting carbon-carbon double bonds (C=C) [33]. The stretching vibrations of the carboxyl group, which are both symmetric and asymmetric, were responsible for the peak at 1569 cm^−1^ [34]. The peak of 1122 cm^−1^ represents C-O-C band intensity. The peak is a C-O stretching vibration at 1070 cm^−1^ [35]. The cross-linking peaks with the C-O-H at 993 cm^−1^ [36].The TEM and FTIR results both proved that DMIPs are attached to CNTs, and CNTs@DMIPs have been successfully synthesized.

### 3.2. Scatchard Analysis

**Figure 3:**
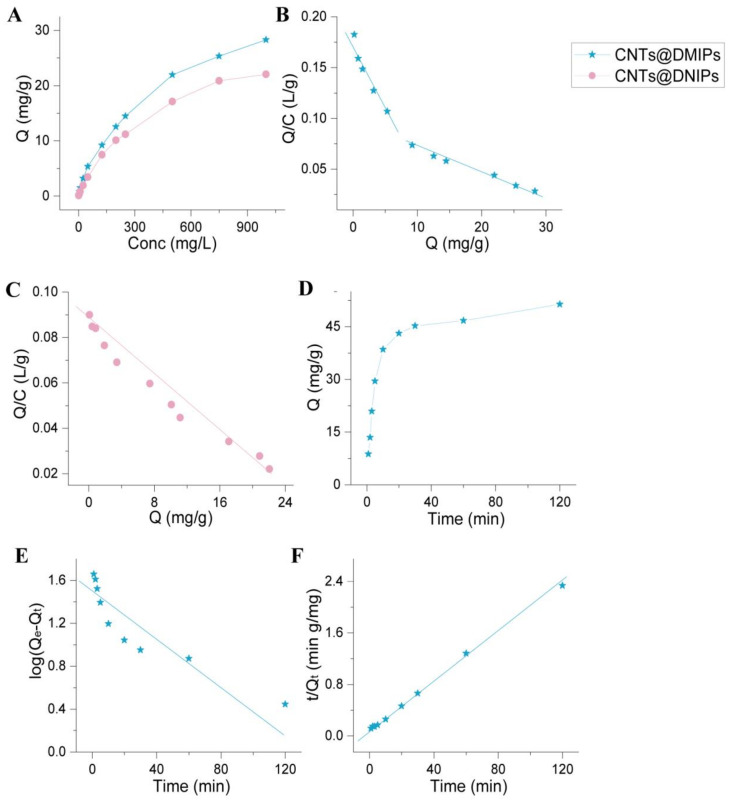
The adsorption isotherm (**A**); Scatchard analysis of DMIPs (**B**) and DNIPs (**C**); The effect of the contact time for the absorption capacity of DMIPs (**D**); The pseudo-first order kinetics (**E**) and the pseudo-second order kinetics (**F**) for DMIPs.

As shown in Figure 3, the isothermal adsorption results showed that molecular imprinting polymer (DMIP) bound to amide herbicide at a higher rate than non-molecular imprinting polymer (DNIP). Scatchard analysis, which evaluates adsorption parameters, was used for the determination of the number of binding sites from Equation (1).

(1) $$\frac{Q}{C}=\frac{Q_{\max}-Q}{K_{d}}$$

where *Q* is the amount of amide herbicide combine with the DMIPs at equilibrium, *C* is the concentration of the free amide herbicide, *Q_max_* is the maximum, *K_d_* is the dissociation constant amount of amide herbicide bound [37]. Calculate the equilibrium amount of amide herbicides bound to DMIPs (Q) according to the plot slope and intercept. Giving *K_d_*, *Q_max_* values. For the DNIPs, the equation of the linear regression was y = −0.0029x + 0.0836, with a *Q_max_* value of 28.83 mg g^−1^. The Scatchard plot showed that there were two kinds of binding sites in the DMIPs with linear regression equations y = −0.0135x + 0.1746, *Q*_*max*1_ = 12.93; y = −0.0022x + 0.0901, *Q*_*max*2_ = 40.95 mg g^−1^, and the *Q_max_* values of DMIPs was 53.88 mg g^−1^, respectively.

### 3.3. Adsorption Kinetics

The kinetic data were analyzed using the pseudo-first order rate equation (Equation (2)) and the pseudo-second order rate equation (Equation (3)). [38]. The pseudo-first-order model yielded *k*_1_ = 0.0092 min^−1^, *q*_*e*1_ = 27.84 mg g^−1^, and R^2^ = 0.8244, while the pseudo-second-order model yielded *k*_2_ = 0.0038 g mg^−1^ min^−1^, *q*_*e*2_ = 52.93 mg g^−1^, and R^2^ = 0.9989. Due to its higher correlation coefficient, the pseudo-second-order model was more suitable for explaining the sorption kinetics of DMIPs on amide herbicides.

(2) $$\log\left(q_{e1}-q_{t}\right)=\log q_{e1}-k_{1}t$$

(3) $$\frac{t}{q_{t}}=\frac{1}{k_{2}q_{e2}^{2}}+\frac{t}{q_{e2}}$$

### 3.4. Selectivity

To measure the adsorption selectivity of the synthesized CNTs@DMIPs toward amide herbicide, a solution containing amide herbicides and other herbicide (atrazine) as a reference was investigated. Table 1 showed that, under optimum experimental conditions, atrazine showed a lower imprinting effect compared with amide herbicides in the CNTs@DMIPs adsorption procedure. The following Equations (4)–(6) were used to calculate the values of the relative selectivity coefficient *β* [39]:

(4) $$\delta =\frac{Q}{C}$$

(5) $$\alpha =\frac{\delta_{\left(\mathrm{amideherbicide}\right)}}{\delta_{\left(\mathrm{atrazine}\right)}}$$

(6) $$\beta =\frac{\alpha_{\mathrm{CNTs}@\mathrm{DMIPs}}}{\alpha_{\mathrm{CNTs}@\mathrm{DNIPs}}}$$

Where *δ*, *Q*, and *C* are the static distribution coefficients, binding amount, and initial solution concentration of amide herbicide and atrazine, respectively. *α* was represented coefficient of selectivity of CNTs@DMIPs or CNTs@DNIPs. The *β* values of amide herbicide were 2.09, 1.27, 1.92, 1.90, 2.60, 2.78, and 1.81, respectively, which might result from the imprinting effect.

### 3.5. Extraction Conditions Optimisation

**Figure 4:**
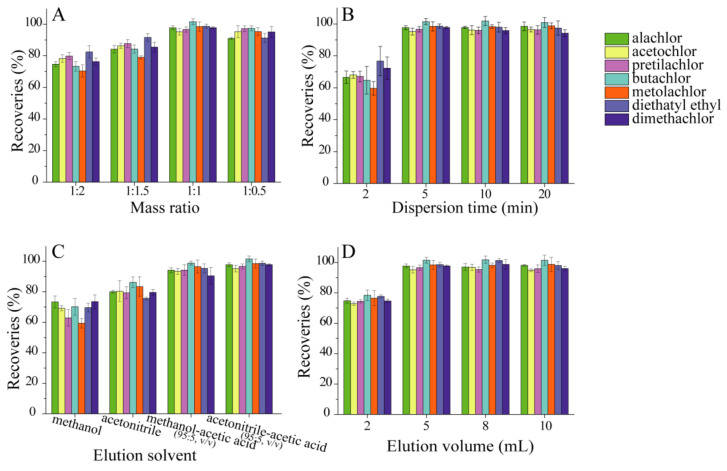
Effects of CNTs@DMIPs mass ratio (**A**); dispersion time (**B**); elution solvent (**C**) and elution volume (**D**) on amide herbicide recoveries.

Influence of mass ratio of CNTs@DMIPs to samples and dispersion time: to facilitate sample transfer to the cartridge and ensure full adsorption of the sample component, a suitable sorbent/sample mass ratio was selected. Our research evaluated variable sorbent/sample proportions (1:2, 1:1.5, 1:1, and 1:0.5) using 200 mg sample (Figure 4A). Heterogeneous mixing of the sample and low recoveries resulted from the lower amount of CNTs@DMIPs sorbent. When the ratio was 1:1, the results revealed that satisfactory recoveries were achieved. Further increasing the ratio showed no improvement in the extracted yield. Therefore, the sorbent/sample proportion of 1:1 was chosen for further investigation.

The dispersion procedure requires the sample to be completely dispersed in the sorbent. Dispersion times of 2, 5, 10, and 15 min have been assessed. As could be seen, an important factor influencing the sorption process was the dispersion time. As seen in Figure 4B, adsorption increased during the first 5 min before remaining constant. In order to achieve satisfactory recoveries, sufficient time was required to reach equilibrium for the adsorption of amide herbicide from the fish samples. It was shown that 5 min gave good recoveries and was considered to be optimal.

The influence of washing and elution conditions: the effect of washing solvent and volume on recoveries and selectivity was explored using 200 mg of CNTs@DMIPs sorbent and 200 mg of fish sample. The water-methanol mixtures (methanol content of 1%, 5%, 10% and 20%) were studied. 10% methanol could be obtained from the satisfied recoveries. Different volumes of 10% methanol (1, 2, 3, 4 mL) were tested for effective washout of interferents with a minimum volume of wash solution. To achieve higher recoveries, 3 mL was finally chosen. Finally, amide herbicides were eluted using methanol, acetonitrile, methanol-acetic acid (95:5, *v*/*v*), and acetonitrile-acetic acid (95:5, *v*/*v*). The acetonitrile/acetic acid (95:5, *v*/*v*) gave the best recoveries (Figure 4C). The optimum volume of acetonitrile-acetic acid (95:5, *v*/*v*) was evaluated with different volumes (2 mL, 5 mL, 8 mL and 10 mL).The outcome demonstrated that the yield of amide herbicides improved as the elution volume increased from 2 to 5 mL, then remained constant even when further increased to 10 mL (Figure 4D). A volume of 5 mL was selected as the optimum elution volume, taking into account the elution efficiency and solvent consumption.

### 3.6. Matrix Effect

When determining amide herbicides in various (complex) matrices, matrix effects are important when using LC-QqQ MS. It can severely impair the reproducibility and accuracy of the method, as well as the quantitative analysis of compounds at trace levels. Four different matrices (including four types of fish) were employed in this matrix study to evaluate this possible effect. To measure the matrix effect, two different test solutions were used (A standard solution in solvent, B standard solution prepared with the extract from a blank fish sample). Area values ratio of B/A between 0.9 and 1.1 showed an acceptable signal strengthening or masking effect, while ratios above 1.1 or below 0.9 indicated a high matrix influence [10]. As calculated, the matrix effect was in the range of 0.93–1.08. It was because of the selectivity of the CNTs@DMIPs and the improved purification effect of amide herbicides. The results demonstrated that the CNTs@DMIPs-MSPD procedure is an effective method for the pre-treatment of samples in reducing the interfering components of the matrix.

### 3.7. Method Validation

To validate the method, the following parameters were used: linearity, limits of detection, quantification and precision. The spiked fish sample was used for the analyses. The linearity of the detector response for the amide herbicide was assessed by injecting a total of seven calibration working standards at concentrations of 0.01, 0.02, 0.05, 0.1, 0.2 0.5 and 1 ng mL^−1^ into the LC-QqQ MS with each concentration repeated three times. A linear relationship was observed between the ratios of the signals of the peak areas and the corresponding concentrations. The method limits of quantification (LOQs) and limits of detection (LODs) were identified as the minimum concentration of the analyte in the fish sample, which, after processing, produces a peak with a signal-noise ratio (S/N) of 3 and to 10, respectively. The recoveries and relative standard deviations (RSDs) were studied by extracting the spiked fish sample (concentration of amide herbicide: 0.05 μg kg^−1^, 1 μg kg^−1^ and 2 μg kg^−1^).The linear range, square correlation coefficient, LOD and LOQ were presented in Table 2. The RSDs were less than 6.55% for intra- and inter-day precision.

### 3.8. Applications to Fish Assays

**Figure 5:**
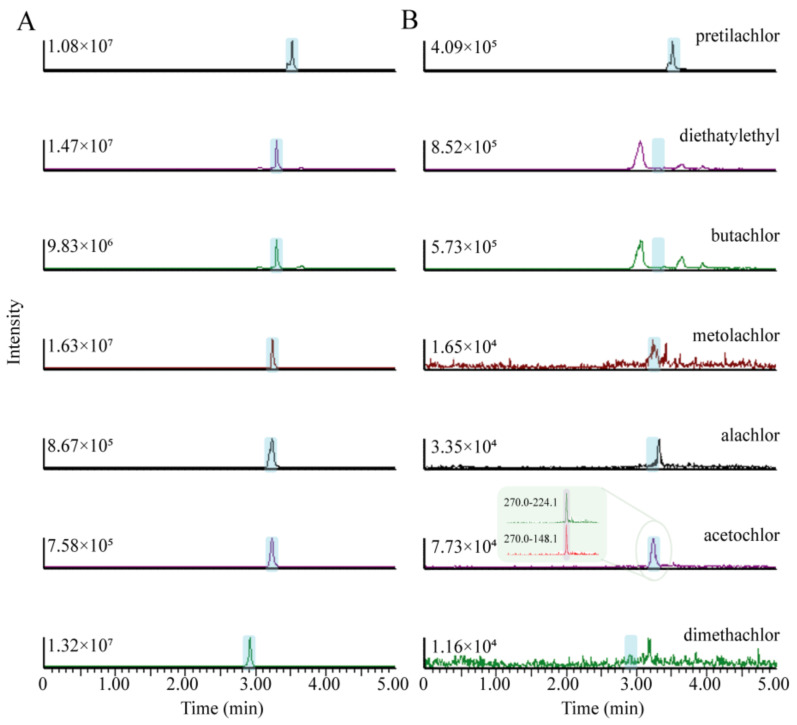
Chromatograms of amide herbicide in (**A**) spiked fish sample, (**B**) one *Silurusasotus* fish sample, and extracted ion chromatogram (**C**) of the acetochlor.

Under the optimised parameters, this method was used successfully to analysis fish samples including four *Cyprinus carpio*, four *Carassius auratust*, four *Hypophthalmichthysmolitrix* and four *Silurusasotus*. In one *Silurusasotus* sample acetochlor was detected in one fish sample (0.83 µg kg^−1^).And the concentrations were found lower than maximum residue level (MRLs) established by GB 23200.57-2016 (National food safety standards-Determination of acetochlor residue in foods) 10 µg kg^−1^ in meat. The chromatograms of spiked fish sample and *Silurusasotus* sample were shown in Figure 5.

Due to the *Silurusasotus* sample with the concentration of acetochlor was 0.83 µg kg^−1^. Ultrasound-assisted extraction was used for extracting the acetochlor from the *Silurusasotus* sample. Unfortunately, there was no signal in acetochlor in 3.23 min. In addition, the CNTs@DMIPs was used as the adsorbent in SPE, the same result was obtained. The reason might be that the concentration was low, and the MSPD was a strong destructive power method. Acetochlor might be residues belongs to the endogenous pollution, deposit for a long time, the traditional ultrasonic assisted extraction method due to its power, it could not be completely within the extracted. 

The amide herbicide was extracted via ultrasonic-assisted extraction. Simultaneously, 200 mg of the synthesized material was loaded into a SPE column and eluted, with the previously developed method yielding negative results. However, positive results can be obtained by using the same adsorbent and matrix solid-phase dispersion as a pretreatment technique. This may be due to two aspects: firstly, the content of amide herbicides in the sample may be small, and secondly, the herbicide residues in the sample may have been deposited for a long time. Additionally, ultrasonic-assisted extraction may not be able to completely extract the internal pollutants due to a lack of ultrasonic power and other factors.

### 3.9. Comparison with Other Methods

We achieved similar or even lower detection limits for amide herbicides using mass spectrometry with different sample preparation methods, as shown in Table 3. Our approach utilized a destructive sample preparation method that improved the extraction of pollutants from fish meat. Moreover, molecular imprinting technology enhanced the extraction selectivity and sensitivity, resulting in lower matrix effects, a cleaner background, and satisfactory detection limits.

Now, these sample preparation procedures are widely used as alternatives to some traditional detection methods, such as extracting samples with high viscosity and poor dispersibility. Similar or lower detection limits are obtained by this method, which can better extract samples with insufficient destructive force, resulting in false negatives. Furthermore, it only requires a smaller sample volume and is also suitable for determining the content of different substances in different parts of the fish body. Even when the fish meat sample is small, a good LOD can be achieved.

## 4. Conclusions

CNTs@MIPs was used as adsorbents to selectively extract amide herbicides from aquatic products by means of MSPD pretreatment. However, the highly destructive manner in which MSPD is embedded in the polymer allows trace residual template molecules to leak out, resulting in false positives in the sample due to template leakage, which has an impact on the results of the quantitative analysis of the sample extraction in actual sample applications. Therefore, in this study, structural analogues of target analytes were selected as dummy templates for the preparation of molecularly imprinted microspheres that provide the unique ability to identify a variety of amide herbicides and eliminate the influence of the tested substances on template leak for both qualitative and quantitative analysis. Subsequently, LC-MS/MS was applied for chromatographic isolation and analysis. Finally, the determination of trace amide herbicides in fishery products was achieved with a satisfactory recovery rate and detection limit. This method has high practical application value and can be employed for the detection and analysis of real samples.

## Figures and Tables

**Table 1:** Selectivity of CNTs@DMIPs.

Recognized Molecule	CNTs@DMIPs		CNTs@DNIPs		*β*
	*δ*	*α*	*δ*	*α*	
alachlor	82.20	10.72	21.57	5.14	2.09
acetochlor	68.33	8.91	29.53	7.03	1.27
pretilachlor	72.80	9.49	20.80	4.95	1.92
butachlor	73.53	9.59	21.17	5.04	1.90
metolachlor	81.70	10.65	17.23	4.10	2.60
diethatylethyl	79.90	10.42	15.73	3.75	2.78
dimethachlor	91.80	11.97	27.77	6.61	1.81
atrazine	7.67	-	4.20	-	-

**Table 2:** The linearity, LOD, LOQ and the recovery of the amide herbicides.

Amide Herbicides	Linearity μgkg^−1^	LOD μgkg^−1^	LOQ μgkg^−1^	Spiked Level 0.05 μgkg^−1^		Spiked Level 1.0 μgkg^−1^		Spiked Level 2.0 μgkg^−1^	
				Recovery (%)	RSD (%)	Recovery (%)	RSD (%)	Recovery (%)	RSD (%)
alachlor	0.05–2.5	0.025	0.05	95.83	4.16	94.68	1.58	94.74	4.52
acetochlor	0.025–2.5	0.0125	0.025	98.90	3.35	93.02	3.31	104.02	5.60
pretilachlor	0.025–2.5	0.0125	0.025	100.70	2.55	96.47	3.77	102.51	4.79
butachlor	0.05–2.5	0.025	0.05	90.74	6.28	87.34	3.97	86.90	4.74
metolachlor	0.025–2.5	0.0125	0.025	94.65	4.36	86.96	3.85	98.93	1.31
diethatyl ethyl	0.05–2.5	0.025	0.05	98.55	6.55	101.47	3.58	100.02	1.58
dimethachlor	0.025–2.5	0.0125	0.025	94.92	6.30	91.12	4.33	93.23	6.14

**Table 3:** Comparison of the proposed method with other reported methods for the extraction and determination of amide herbicides.

Extraction	Clean-Up	Determination	Sample	Analytical	LOD (μgkg^−1^)	Reference
Dispersive liquid-liquid microextraction	QuEChERS	GC-MS	Oilseeds	Alachlor, acetochlor	0.07–0.7	[5]
Dispersive SPE	SPE	LC-MS/MS	Honey	Metolachlor	0.1	[6]
Shaking	QuEChERS	LC-MS/MS	Chicken eggs	Butachlor, acetochlor	0.2	[7]
Shaking	SPE	LC-MS	Cereal grain	Metazachlor	0.08	[8]
Dispersive SPE	Multiplug filtration clean-up.	GC-MS/MS	Leeks	Alachlor, butachlor, acetochlor	1.7–2.6	[9]
Reversed-dispersive SPE	Multiplug filtration clean-up.	LC-MS/MS	Peanut	Acetochlor	2.0	[10]
MSPD	CNTs@DMIPs	LC-MS/MS	Fish	Alachlor, acetochlor, pretilachlor, butachlor, metolachlor, diethatyl ethyl, dimethachlor	0.0125– 0.025	Proposed method

## Data Availability

Not applicable.
